# Venous outflow time profiles: promising imaging biomarkers for futile recanalization in acute ischemic stroke due to large vessel occlusion

**DOI:** 10.3389/fneur.2025.1714681

**Published:** 2026-01-14

**Authors:** Hansheng Wang, Jingjie Wang, Weidong Fang, Zhiwei Zhang, Li Tong, Yongmei Li

**Affiliations:** Department of Radiology, The First Affiliated Hospital of Chongqing Medical University, Chongqing, China

**Keywords:** large vessel occlusion, acute ischemic stroke, endovascular thrombectomy, four-dimensional CT angiography, venous outflow, futile recanalization

## Abstract

**Background:**

To investigate whether venous outflow (VO) profiles derived from four-dimensional computed tomographic angiography (4D-CTA) correlate with futile recanalization (FR).

**Method:**

This study consecutively enrolled acute ischemic stroke patients with large vessel occlusion (AIS-LVO) who underwent endovascular thrombectomy (EVT) and achieved successful recanalization (modified thrombolysis in cerebral infarction [mTICI] score of 2b–3). An unfavorable functional outcome was defined as a modified Rankin Scale (mRS) score of 3–6 at 90 days after EVT. VO profiles were assessed using 4D-CTA, including the cortical vein opacification score (COVES) and three cortical venous filling (CVF) time points per hemisphere: initial opacification (CVF1), optimal opacification (CVF2), and complete outflow (CVF3). Intervals between the time points CVF21 and CVF31 and bilateral CVF time differences (rCVFs) were calculated. A binary logistic regression analysis was performed to identify independent predictors of FR.

**Results:**

A total of 167 AIS-LVO patients were enrolled, with 84 patients in the favorable outcome group (FOG, mRS 0–2) and 83 patients in the futile recanalization group (FRG, mRS 3–6). Compared with patients in FOG, patients in FRG were older, had higher National Institutes of Health Stroke Scale (NIHSS) scores, a greater prevalence of atrial fibrillation, lower Alberta Stroke Program Early CT Score (ASPECTS), and prolonged rCVF3 and rCVF31 (all *p* and FDR-corrected *p* < 0.1). A multivariable analysis identified age (adjusted odds ratio [aOR] = 1.06, 95% CI = 1.03–1.10, *p* = 0.001), ASPECTS (aOR = 0.73, 95% = CI 0.59–0.89, *p* = 0.002), and rCVF31 (aOR = 1.26, 95% CI = 1.04–1.52, *p* = 0.016) as independent predictors of FR.

**Conclusion:**

In addition to older age and lower baseline ASPECTS, prolonged ischemic-side venous duration (quantified by rCVF31) can independently predict FR. These findings suggest that VO parameters serve as novel imaging biomarkers for outcome stratification in AIS-LVO patients undergoing EVT.

## Introduction

Compared with intravenous thrombolysis (IVT), endovascular thrombectomy (EVT) significantly improves the functional independence of anterior circulation acute ischemic stroke due to large vessel occlusion (AIS-LVO) (1–3). With the aid of computed tomography perfusion (CTP), the treatment time window for EVT has been extended from the early (<6 h) to the expanded (6–24 h) window (4, 5). However, recent studies have reported that nearly half of AIS-LVO patients experience unfavorable functional outcomes (modified Rankin Scale [mRS] 3–6) despite successful recanalization (modified Thrombolysis in Cerebral Infarction [mTICI] 2b–3) after EVT, which is known as futile recanalization (FR) (2, 6–8). Reported independent predictors of FR included older age, higher National Institutes of Health Stroke Scale (NIHSS) score on admission, symptomatic intracerebral hemorrhage (sICH), lower Alberta Stroke Program Early CT Score (ASPECTS), poor collaterals, and absent penumbra (9–12). However, studies on imaging biomarkers for FR remain limited, warranting the need for more investigation.

Recent studies suggest that venous outflow (VO) profiles may reflect collateral blood flow and tissue microperfusion in AIS-LVO (13, 14). VO profile studies have primarily focused on the degree and speed of cortical venous opacification. The degree of venous opacification can be semi-quantitatively assessed using the cortical vein opacification score (COVES), which has been associated with edema, first-pass reperfusion during EVT, early neurological improvement, long-term functional outcomes, and FR (15–19). In contrast, the assessment of cortical venous opacification speed varies across studies, and the association between VO time profiles and FR remains unclear (20–26). Moreover, no prior studies have combined COVES with quantitative VO time profiles to examine their association with FR using four-dimensional computed tomographic angiography (4D-CTA) in a relatively large cohort. We hypothesized that VO profiles assessed with 4D-CTA could serve as novel imaging biomarkers for predicting FR in anterior circulation AIS-LVO patients.

## Methods

### Patients

This study was approved by the Ethics Committee of The First Affiliated Hospital of Chongqing Medical University (2025-296-01) and followed the guidelines of the Declaration of Helsinki. Patient-informed consent was waived by the review boards for this retrospective study. Consecutive AIS-LVO patients who visited the stroke center of our hospital between January 2021 and April 2025 were reviewed in this retrospective study. The inclusion criteria of patients were as follows: (1) age ≥18 years; (2) presence of an anterior circulation LVO involving internal carotid artery (ICA) or the M1 or M2 segment of the middle cerebral artery; (3) initiation of EVT within 24 h after symptom onset, with or without prior intravenous thrombolysis; (4) successful recanalization (mTICI 2b–3) following EVT; (5) having undergone non-contrast computed tomography (NCCT) and 4D-CTA scanning before EVT; and (6) availability of mRS at 3 months after EVT. The exclusion criteria include patients with a history of stroke prior to the current admission, with the presence of an identifiable post-stroke cyst (encephalomalacia) on NCCT, bilateral AIS, and posterior circulation AIS, and with poor image quality for analysis. Patients with cortical vein opacification at the final scan time point were also excluded to avoid incomplete time-interval assessment. A total of 15 patients were excluded based on this criterion.

Demographic and clinical data were reviewed from the hospital information system (HIS), including age, sex, risk factors (such as hypertension, diabetes, hyperlipidemia, and smoking), and NIHSS score on presentation. sICH was determined according to Heidelberg criteria (27) and a trained physician assessed the mRS via standardized telephone interview.

Patients were divided into a favorable outcome group (FOG, mRS 0–2) and a futile recanalization group (FRG, mRS 3–6) based on mRS at 90 days after EVT.

### Imaging protocols

The included patients underwent a one-stop multimodal CT scan, including NCCT and 4D-CTA performed on a 320-row detector CT scanner (Aquilion ONE, Canon Medical Systems Corporation, Otawara, Japan), as previously described (24, 28). Briefly, the 4D-CTA consisted of 19 whole-brain volume packages, with 1 non-contrast package prior to contrast injection and 18 packages after contrast injection. All datasets were transferred to the Vitrea workstation (version 4.0.693, Vital Images) for the reconstruction of three-dimensional maximum intensity projection (3D-MIP) images, including 3D-MIP CT angiography (CTA) and 3D-MIP CT venography (CTV) images, which could dynamically demonstrate the arterial and venous opacification process. Each 3D-MIP CTA/CTV image had a time point annotation.

### Imaging analysis

**Figure 1:**
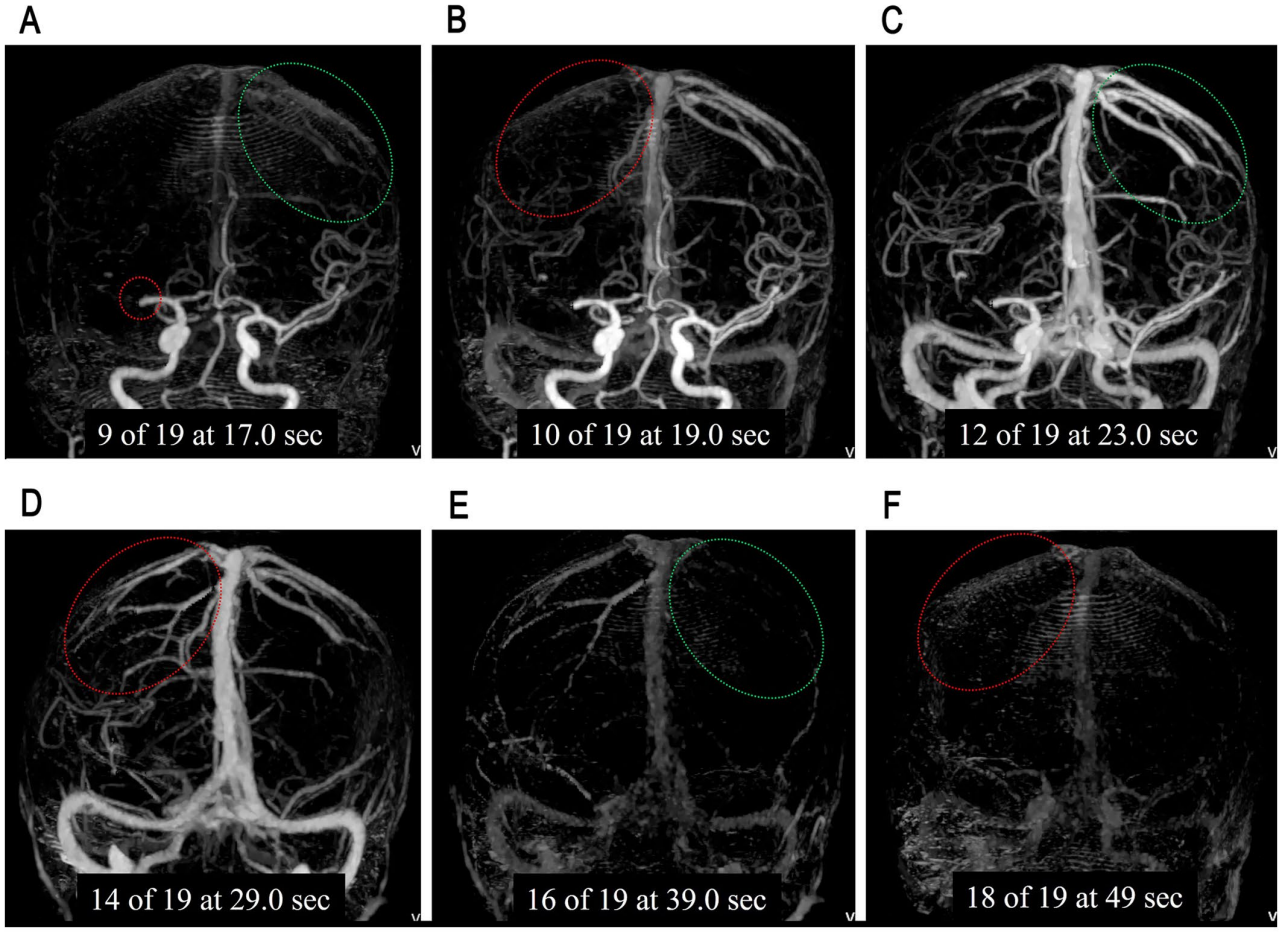
An example of bilateral cortical venous filling (CVF) time points. **(A)** Showed the occlusion of the first segmentation of the right middle cerebral artery (red circle), with the cortical veins beginning to opacify in the normal side hemisphere (NS-CVF1, green circle). **(B)** Showed the cortical veins started to opacify in the ischemic side cortical veins (IS-CVF1, red circle). **(C)** Showed the optimal cortical veins opacification in the normal side hemisphere (NS-CVF2, green circle). **(D)** Showed the optimal cortical veins opacification in the ischemic side hemisphere (IS-CVF2, red circle). **(E)** Showed the disappearance of contrast in the normal side cortical veins (NS-CVF3, green circle). **(F)** Showed the disappearance of contrast in the ischemic side cortical veins (IS-CVF3, red circle).

By reviewing contrast enhancement of all cortical veins draining into the superior sagittal sinus on 3D-MIP CTV images, three cortical venous filling (CVF) time points were recorded for each hemisphere (23, 29): CVF1 was the time point when any cortical vein began to opacify, CVF2 was the time point when most cortical veins showed optimal opacification, and CVF3 was the time point when contrast completely outflowed and all cortical veins were no longer opacified. The identification of bilateral CVF time points is shown in Figure 1. The VO time profiles were acquired by calculating the time intervals between CVF2 and CVF1 (CVF21), CVF3, and CVF1 (CVF31) for each hemisphere, and the CVF time differences between ischemic side hemisphere and normal side hemisphere (ischemic side CVFs minus normal side CVFs) were presented as rCVF1, rCVF2, rCVF3, rCVF21, and rCVF31.

COVES was applied to evaluate the opacification degree of the main cortical outflow veins on the ischemic side. First, the peak venous phase images were identified based on the venous time attenuation curve. Second, the opacification degree of sphenoparietal sinus (SPS), superficial middle cerebral vein (SMCV), and vein of Labbé (VOL) on the ischemic side was compared with that of the corresponding veins on the normal side on peak venous phase images, and the opacification degree of each vein was assigned a score ranging from 0 to 2: 0 for no opacification, 1 for moderate opacification, and 2 for full opacification (30, 31). Finally, the sum of the three venous scores constituted COVES. CVFs and COVES were independently determined by two experienced doctors, who were blinded to clinical data.

ASPECTS was determined on baseline NCCT images (32). The American Society of Interventional and Therapeutic Neuroradiology/Society of Interventional Radiology (ASITN/SIR) collateral grading system was used for arterial collateral scoring (33). ASPECTS and ASITN/SIR collateral grades were assessed by consensus reading of two experienced doctors blinded to clinical data.

### Statistical analysis

Inter-rater reliability for COVES and CVFs was assessed using the intraclass correlation coefficient (Supplementary material). Continuous variables were reported as median and interquartile range (IQR) and compared between groups with the Wilcoxon rank-sum test. Categorical variables were represented as frequencies and percentages and compared using the chi-square test. *p-*values for group comparisons of imaging metrics were corrected using the FDR method. Given the exploratory nature of this study and the aim to comprehensively identify all potential prognostic variables for inclusion in the subsequent multivariable model, this study adopted a relatively liberal significance threshold (*p* < 0.1 or FDR-corrected *p* < 0.1) for variable selection. All variables meeting this criterion were entered into a binary logistic regression model for further investigation. Besides, we forced the inclusion of key confounders known to influence outcomes, irrespective of their univariate *p*-values. These pre-specified variables included occlusion site, ASITN/SIR collateral grade, COVES, and mTICI grade. A binary logistic regression analysis was performed using the forced entry method to identify independent predictors of FR. The results were presented as odds ratios (ORs) with 95% confidence intervals (CIs), and statistical significance was defined as a *p*-value of < 0.05. All analyses were conducted using IBM SPSS Statistics (version 26.0).

## Results

A total of 167 patients were finally enrolled in this study, with 84 patients (50.3%) in FOG and 83 patients (49.7%) in FRG. A post-hoc power analysis showed that the achieved statistical power was 0.89. The intraclass correlation coefficient of CVFs and COVES were all >0.8 (see Supplementary material).

Compared with patients in FOG, those in FRG were significantly older (71 [IQR 66, 79] vs. 63.5 [IQR 53, 73], *p* < 0.001), had higher NIHSS on admission (14 [IQR 10, 18] vs. 10 [IQR 6, 14], *p* < 0.001), and had higher frequency of atrial fibrillation (42.2% vs. 26.2%, *p* = 0.029). Complete demographic and clinical data are shown in Table 1.

**Table 1:** Demographics and clinical data of FOG and FRG.

Clinical variables	All patients, *N* = 167	FOG, *N* = 84	FRG, *N* = 83	*p*
Female, *N* (%)	68/167 (40.7%)	30/84 (35.7%)	38 (45.8%)	0.185
Age, years, median (IQR)	69 (58, 77)	63.5 (53, 73)	71 (66, 79)	<0.001
Presentation NIHSS, median (IQR)	12 (8, 17)	10 (6, 14)	14 (10, 18)	<0.001
Time from symptom onset to admission (h)				
*t* ≤ 6 h, *N* (%)	113/167 (67.6%)	59/84 (35.3%)	54/83 (32.3%)	0.638
6 < *t* ≤ 24 h, *N* (%)	54/167 (32.4%)	25/84 (15.0%)	29/83 (17.4%)	0.586
Hypertension, *N* (%)	106/167 (63.5%)	49/84 (58.3%)	57/83 (68.7%)	0.165
Diabetes, *N* (%)	55/167 (32.9%)	23/84 (27.4%)	32/83 (38.6%)	0.125
Coronary heart disease, *N* (%)	29 (17.4%)	13/84 (15.5%)	16/83 (19.3%)	0.517
Atrial fibrillation, *N* (%)	57/167 (34.1%)	22/84 (26.2%)	35/83 (42.2%)	0.029
Hyperlipidemia, *N* (%)	58/167 (34.7%)	31/84 (36.9%)	27/83 (32.5%)	0.553
Smoking, *N* (%)	65/167 (38.9%)	38/84 (45.2%)	27/83 (32.5%)	0.092
Alcohol, *N* (%)	61/167 (36.5%)	29/84 (34.5%)	32/83 (38.6%)	0.589
IVT, *N* (%)	50/167 (29.9%)	30/84 (35.7%)	20/83 (24.1%)	0.101
mTICI 3, *N* (%)	140/167 (83.8%)	74/84 (88.1%)	66/83 (79.5%)	0.132
sICH	5/167 (3%)	1/84 (1.2%)	4/83 (4.8%)	0.180^*^

FOG, Favorable outcome group; FRG, Futile recanalization group; IQR, Interquartile range; IVT, Intravenous thrombolysis; mTICI, Modified Thrombolysis in Cerebral Infarction; NIHSS, National Institutes of Health Stroke Scale; sICH, Symptomatic intracerebral hemorrhage.

* Fisher’s exact test.

FRG patients also had significantly lower ASPECTS (7 [IQR 5, 8] vs. 8 [IQR 7, 9], FDR-corrected *p* < 0.001) and prolonged VO time profiles, including rCVF3 (10 [IQR 7, 13] vs. 10 [IQR 5, 10], FDR-corrected *p* = 0.019) and rCVF31 (6.4 [IQR 4, 8] vs. 5.4 [IQR 3, 8], FDR-corrected *p* = 0.082). Full imaging metrics are detailed in Table 2.

**Table 2:** Wilcoxon rank-sum test of imaging metrics between FOG and FRG.

Imaging metrics	All *N* = 167	FOG *N* = 84	FRG *N* = 83	*p*	FDR-corrected *p* value
ASPECTS	8 (6, 9)	8 (7, 9)	7 (5, 8)	<0.001	<0.001
Occlusion site				0.356^*^	0.564
ICA	45	21	24		
M1	104	51	53		
M2	18	12	6		
COVES	3 (2, 5)	4 (3, 5)	3 (2, 4)	0.034	0.129
ASITN/SIR collateral grade	3 (2, 4)	3 (2, 4)	2 (2, 4)	0.397	0.539
IS-CVF1	19.6 (16.6, 23.2)	19.0 (16.6, 23.2)	20.6 (16.6, 23.2)	0.241	0.509
IS-CVF2	29.0 (23.2, 33.0)	29.0 (23.2, 33)	29.2 (25.1, 33.0)	0.187	0.508
IS-CVF3	48.0 (43.0, 53.0)	48.0 (42.2, 49.0)	48.0 (43.0, 53.0)	0.030	0.143
IS-CVF21	8.6 (7.0, 10.6)	8.6 (7.0, 10.0)	8.6 (6.6, 10.6)	0.846	0.946
IS-CVF31	26.0 (23.0, 29.9)	25.6 (23, 29.8)	26.7 (24.4, 30.4)	0.136	0.431
NS-CVF1	16.6 (14.2, 19.6)	16.6 (14.0, 19.2)	17.5 (14.6, 19.6)	0.375	0.548
NS-CVF2	23.2 (20.6, 27.2)	23.2 (20.7, 27.2)	25.0 (20.6, 27.2)	0.330	0.570
NS-CVF3	38.0 (33.0, 43.0)	38.0 (33.0, 43.0)	38.0 (33.0, 43.0)	0.909	0.909
NS-CVF21	7.6 (6.0, 8.6)	7.3 (6.0, 8.6)	7.6 (6.0, 8.6)	0.695	0.825
NS-CVF31	20.4 (17.4, 24.0)	20.4 (17.7, 23.9)	20.8 (16.6, 24.0)	0.694	0.879
rCVF1	2.6 (2.0, 4.0)	2.3 (1.9, 4.0)	3.0 (2.0, 4.0)	0.197	0.468
rCVF2	4.0 (2.6, 6.0)	4.0 (2.0, 6.0)	4.2 (2.6, 6.0)	0.294	0.559
rCVF3	10.0 (5.0, 10.0)	10.0 (5.0, 10.0)	10.0 (7.0, 13.0)	0.002	0.019
rCVF21	1.8 (0.0, 2.6)	1.7 (0.0, 2.5)	1.8 (0.0, 2.6)	0.865	0.913
rCVF31	6.0 (3.0, 8.0)	5.4 (3.0, 8.0)	6.4 (4.0, 8.0)	0.013	0.082

* The *p*-value was determined by the Fisher–Freeman–Halton exact test.

ASITN/SIR, American Society of Interventional and Therapeutic Neuroradiology/Society of Interventional Radiology; ASPECTS, Alberta Stroke Program Early CT Score; COVES, Cortical vein opacification score; CVF, Cortical venous filling; FOG, Favorable outcome group; FRG, Futile recanalization group; ICA, Internal carotid artery; IQR, Interquartile range; IS, Ischemic side; M1, The first segment of the middle cerebral artery; M2, The second segment of the middle cerebral artery; NS, Normal side.

A binary logistic regression analysis identified age, ASPECTS, and rCVF31 as independent predictors of FR (all *p* < 0.05). Specifically, older age (adjusted odds ratio [aOR] = 1.06, 95% CI = 1.03–1.10, *p* = 0.001) and prolonged rCVF31 (aOR = 1.26, 95% CI = 1.04–1.52, *p* = 0.016) were risk factors of FR, whereas ASPECTS was a significant protective factor. For each one-point increase in ASPECTS, the odds of FR decreased by 27% (aOR = 0.73, 95% CI = 0.59–0.89, *p* = 0.002). All logistic regression results are presented in Table 3.

**Table 3:** Independent clinical and imaging predictors for FR.

Predictors	aOR	95% CI	*p*
Age	1.06	1.03–1.10	0.001
NIHSS on presentation	1.06	0.99–1.13	0.074
Atrial fibrillation	1.46	0.63–3.40	0.376
Smoking	0.57	0.25–1.27	0.168
sICH	1.48	0.13–17.00	0.754
ASPECTS	0.73	0.59–0.89	0.002
Occlusion site			
ICA	1.00 (reference)		
M1	0.83	0.34–2.03	0.683
M2	0.41	0.10–1.66	0.211
mTICI			
mTICI 2b	1.00 (reference)		
mTICI 3	0.40	0.14–1.19	0.100
ASITN/SIR	1.29	0.89–1.87	0.183
COVES	1.03	0.80–1.32	0.801
rCVF3	0.96	0.82–1.12	0.606
rCVF31	1.26	1.04–1.52	0.016
Constant	0.04		0.044

aOR, Adjusted odds ratio; ASITN/SIR, American Society of Interventional and Therapeutic Neuroradiology/Society of Interventional Radiology; ASPECTS, Alberta Stroke Program Early CT Score; CI, Confidence interval; COVES, Cortical vein opacification score; FR, Futile recanalization; ICA, Internal carotid artery; M1, The first segment of the middle cerebral artery; M2, The second segment of the middle cerebral artery; mTICI, Modified Thrombolysis in Cerebral Infarction; NIHSS, National Institutes of Health Stroke Scale, rCVF3, Relative difference in CVF3 between the ischemic side hemisphere and the normal side hemisphere; rCVF31, Relative time difference of the whole venous phase between the ischemic side hemisphere and the normal side hemisphere; sICH, Symptomatic intracerebral hemorrhage.

## Discussion

This study demonstrated that prolonged VO time profiles based on pretreatment 4D-CTA (particularly rCVF31) were independent predictors of FR in anterior circulation AIS-LVO, alongside older age and lower baseline ASPECTS as risk factors for FR. The incidence of FR was 49.7%, consistent with previous studies (9, 12, 19).

Prolonged VO time profiles may represent promising imaging biomarkers of FR in AIS-LVO. In this study, prolonged rCVF3 and rCVF31 indicated slower VO speed in the ischemic side hemisphere compared with the normal side hemisphere. Moreover, only the prolonged entire venous duration (rCVF31) in the ischemic side hemisphere was identified as an independent predictor of FR. The strength of this study lies in the fact that it is focused on VO time profiles in the ischemic side and their association with FR in a relatively large cohort. Su et al. reported that a prolonged peak time of the superior sagittal sinus (SSS) was identified as an independent predictor of FR (21). However, the SSS peak time included the time interval from scan initiation to venous contrast arrival, which may be influenced by the trigger timing of scanning. Recently, prolonged venous transit (PVT), which is defined as a time to maximum of residue function (Tmax) ≥ 10 s within the posterior SSS or torcula, has been reported to be associated with worse neurological improvement, poorer 90-day functional outcomes, and higher odds of mortality in AIS-LVO patients who achieved reperfusion (34–37). However, VO time profiles of SSS represent the whole cerebral cortical venous outflow, which is not the case in the ischemic hemisphere. In contrast to previous research that employed heterogeneous assessment methods and reported an association between prolonged VO time profiles and unfavorable clinical outcomes in AIS-LVO patients receiving broader treatment measures (e.g., IVT or conservative therapy) (22, 24, 26, 29), the present study adopted stricter inclusion criteria to specifically assess the relationship between VO time profiles on the ischemic side and FR.

Prolonged VO time profiles may indicate microcirculation failure, potentially related to brain tissue edema and microthrombi formation (38). Brain tissue edema leads to elevated interstitial pressure and increased capillary flow resistance in the hypoperfusion area, which may hamper blood flow transit through ischemic brain tissue into the draining veins (39). Besides, studies on “no-reflow” phenomenon suggest that complex factors lead to microthrombi formation, microvascular compression, and contraction (40–42). Brain tissue edema and these pathophysiological alterations collectively lead to microcirculation failure, which impedes blood flow from the artery to the outflow veins despite macrovascular recanalization. For patients with prolonged VO time profiles, hospital treatment and long-term rehabilitation strategies may require optimization. A comprehensive treatment approach addressing microcirculation failure and impaired venous outflow warrants further investigation, with careful evaluation of its associated risks and benefits.

Previous studies have suggested that lower COVES may be an independent predictor of FR or unfavorable outcomes (14, 19, 29, 43, 44). In this study, although a nominally significant difference was observed in COVES between groups in the univariate analysis (*p* = 0.034), this finding was not robust after false discovery rate correction (FDR-corrected, *p* = 0.129), indicating that the observed association may be marginal and should be interpreted with caution. Besides, in the multivariable model adjusted for covariates, COVES was not identified as a significant independent predictor of FR (aOR = 1.03, 95% CI = 0.80–1.32, *p* = 0.801). This discrepancy may result from multiple factors. First, the inclusion criteria varied between studies, including differences in the time window for EVT as well as in the treatment measures administered. Second, most of previous studies acquired COVES on single-phase CTA. The COVES in our study were acquired on peak venous phase images, which allowed full opacification of cortical veins compared with single-phase CTA (45). Finally, some studies did not include both COVES and VO time profiles in the logistic regression analysis simultaneously. The loss of significance of COVES in the multivariable model suggests that it may not provide an independent predictive value for FR beyond age, ASPECTS and VO time profiles in this study.

ASPECTS has been associated with baseline clinical stroke severity, hemorrhagic transformation, cerebral edema, final infarct volume, and 90-day outcomes (46–48). In this study, lower ASPECTS is an independent predictor of FR. Lower ASPECTS may indicate more extensive disruption of the blood–brain barrier and irreversible injury of the brain parenchyma (49, 50). The benefit of EVT in patients with ASPECTS <5 was reduced when weighed against the higher risks (51, 52). Even if successful recanalization is achieved within the time window, more extensive brain tissue injury is associated with more severe edema, microcirculation failure, and higher odds of hemorrhage transformation; these pathophysiological processes may collectively lead to an unfavorable outcome.

Along with previous studies, this study also demonstrated that older AIS-LVO patients were more likely to experience unfavorable outcomes by 90 days despite successful recanalization (2, 12, 53, 54). The potential reasons could be altered metabolism, limited rehabilitation potential, comorbidities, lower medication compliance, and higher prevalence of reperfusion hemorrhage (9, 50, 55, 56). Although the odds of favorable outcomes decreased with age, convincing studies have demonstrated the benefit of EVT for octogenarians (56, 57). Reasonable assessments can identify older AIS-LVO patients who are suitable for EVT.

Despite its well-established role as a powerful predictor of poor outcome, sICH was not an independent prognostic factor in our cohort. The most plausible explanation for this discrepancy is the remarkably low incidence of sICH (1 in FOG, 4 in FRG) in our study, which resulted in limited statistical power to detect a significant association.

The ASITN/SIR collateral grade showed no significant difference between FOG and FRG. It was not an independent predictor of FR in this study. The association between pial arterial collaterals and outcomes remains controversial. Studies have suggested that pial arterial collaterals only reflect the blood supply after LVO; therefore, a more comprehensive assessment of blood circulation, namely the cerebral collateral cascade model, may be a promising approach to study the association between blood circulation and edema, hemorrhagic transformation and clinical outcomes of AIS-LVO (8, 13, 58).

This study, however, has some limitations. This was a single-center retrospective study. The exclusion of patients who had incomplete clinical and imaging data may introduce selection bias. The findings of this study should be applied cautiously. Multicenter studies with a larger population may improve the generalizability and allow for subgroup analyses. Besides, this study only evaluated cortical veins. As an important drainage component of venous outflow, deep veins should be included to provide a more complete picture of cerebral venous hemodynamics and to validate the generalizability of the proposed biomarkers.

## Conclusion

Prolonged ischemic-side venous duration (quantified by rCVF31) independently predicted futile recanalization, in addition to established risk factors such as older age and lower baseline ASPECTS. These findings suggest that VO time profiles may represent novel imaging biomarkers for outcome stratification.

## Data Availability

The original contributions presented in the study are included in the article/Supplementary material, further inquiries can be directed to the corresponding author.
