# Supplementary material for: Venous outflow time profiles: promising imaging biomarkers for futile recanalization in acute ischemic stroke due to large vessel occlusion
Source: Front Neurol. 2026 Jan 14;16:1714681. doi: 10.3389/fneur.2025.1714681 (PMC12846930; doi:10.3389/fneur.2025.1714681)

Supplementary Material

# Supplementary table

Post-hoc inter-rater reliability for COVES and CVFs.

| Indicators | ICC | 95% Confidence Interval | *p*-value |
| --- | --- | --- | --- |
| COVES | 0.843 | 0.724 - 0.914 | < 0.001 |
| Ischemic Side CVF1 | 0.976 | 0.938 - 0.982 | < 0.001 |
| Ischemic Side CVF2 | 0.967 | 0.938 - 0.982 | < 0.001 |
| Ischemic Side CVF3 | 0.871 | 0.770 - 0.930 | < 0.001 |
| Normal Side CVF1 | 0.974 | 0.950 - 0.986 | < 0.001 |
| Normal Side CVF2 | 0.970 | 0.943 - 0.984 | < 0.001 |
| Normal Side CVF3 | 0.961 | 0.898 - 0.983 | < 0.001 |

**Note:** Another trained rater, blinded to the initial clinical and imaging data, independently determined COVES and CVFs according to previous criteria to a randomly selected subset of 40 patients (approximately 24% of the entire cohort). The inter-rater reliability for COVES and CVFs were assessed by intraclass correlation coefficient (ICC (2,1)).

**Abbreviations:** COVES, cortical vein opacification score; CVF, cortical venous filling; ICC, intraclass correlation coefficient

# Supplementary figure

Post-hoc power analysis


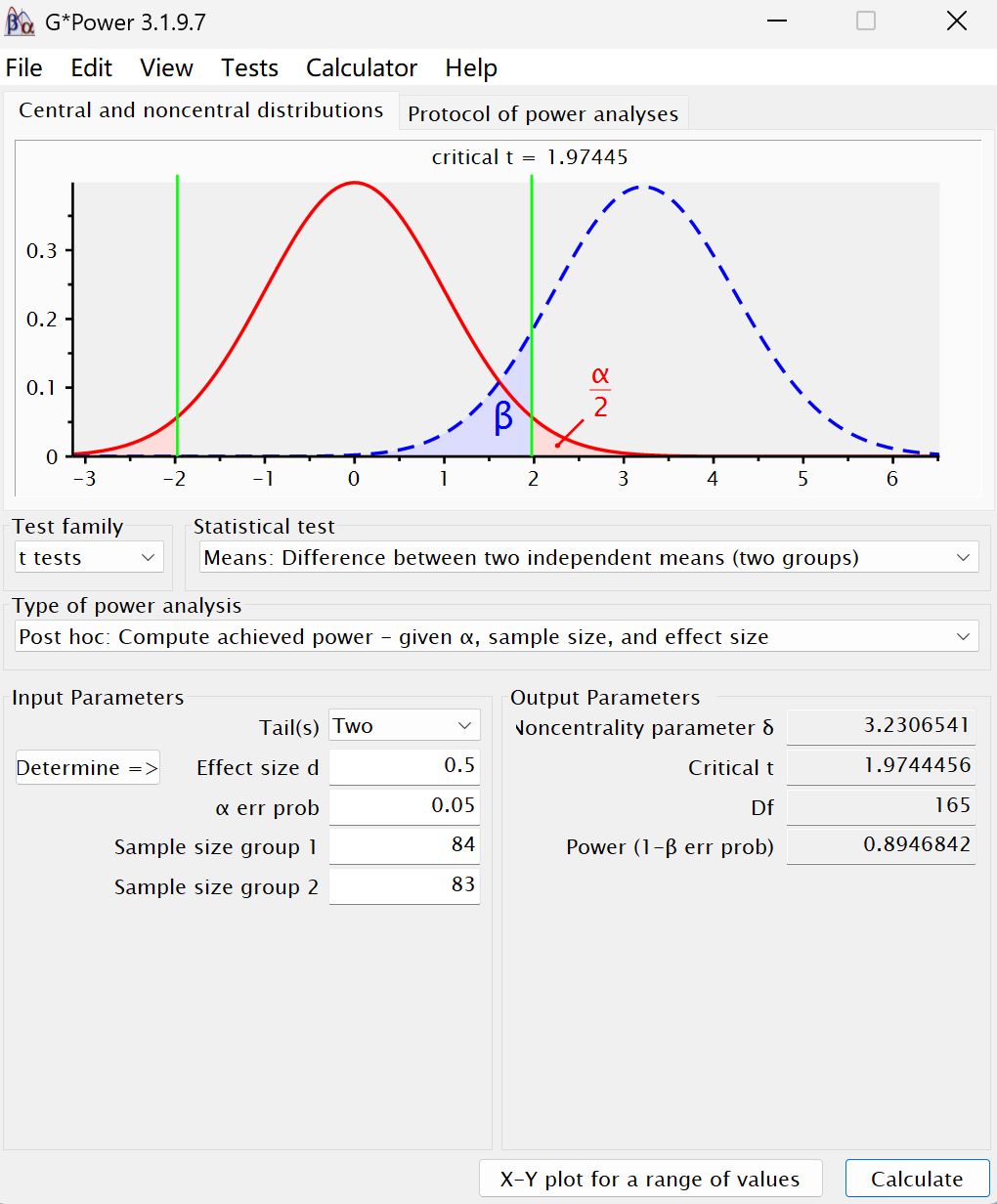

Supplement: Supplementary file 1 [file Supplementary_file_1.docx]
